# *anti*-2,2,3,3,6,6,7,7,10,10,11,11,14,14,15,15-Hexadeca­methyl-2,3,6,7,10,11,14,15-octa­silapenta­cyclo­[10.4.2.2^4,9^.0^5,8^.0^13,16^]icosa-1(17),4,8,12(18),13(16),19-hexa­ene

**DOI:** 10.1107/S1600536813002584

**Published:** 2013-02-06

**Authors:** Yuki Toma, Kyohei Otsuka, Soichiro Kyushin

**Affiliations:** a Department of Chemistry and Chemical Biology, Graduate School of Engineering, Gunma University, Kiryu, Gunma 376-8515, Japan

## Abstract

The title compound, C_28_H_52_Si_8_, was synthesized by condensation of two mol­ecules of 1,2,3,4-tetra­kis­(chloro­dimethyl­sil­yl)benzene with lithium. The 3,4-disila-1,2-benzocyclo­butene rings in the centrosymmetric molecule are bridged by 1,1,2,2-tetra­methyl­disilanylene chains with an *anti* conformation. The benzene rings are deformed by fusion with a 3,4-disilacyclo­butene ring resulting in a slight boat conformation. Two Si—C bonds are bent to reduce the steric repulsion between the methyl groups on the two Si atoms and the methyl groups on another two Si atoms.

## Related literature

For structures of cyclo­phanes bridged by tetra­methyl­disilanylene chains, see: Sakurai *et al.* (1986 ▶); Sekiguchi *et al.* (1989 ▶).
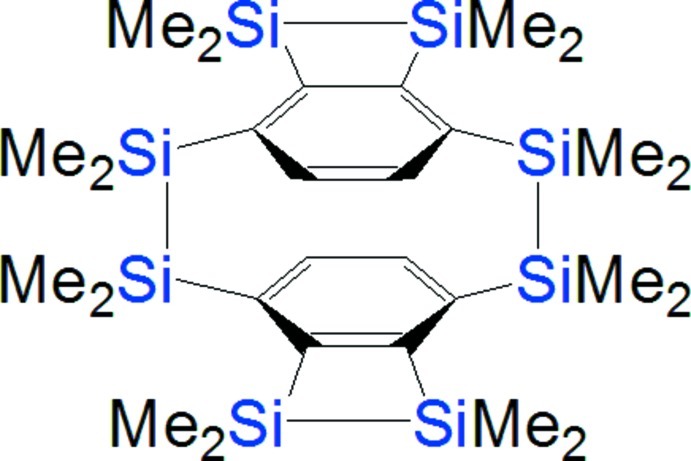


## Experimental

### 

#### Crystal data

C_28_H_52_Si_8_
*M* *_r_* = 613.42
Monoclinic, 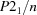

*a* = 7.9801 (8) Å
*b* = 18.9087 (14) Å
*c* = 12.6222 (10) Å
β = 104.2788 (9)°
*V* = 1845.8 (3) Å^3^
*Z* = 2
Mo *K*α radiation
μ = 0.31 mm^−1^
*T* = 203 K
0.25 × 0.25 × 0.25 mm

#### Data collection

Rigaku R-AXIS IV Imaging Plate diffractometer
Absorption correction: multi-scan (*REQAB*; Jacobson, 1998 ▶) *T*_min_ = 0.927, *T*_max_ = 0.927
9672 measured reflections
3096 independent reflections
3076 reflections with *I* > 2σ(*I*)
*R*_int_ = 0.016

#### Refinement

*R*[*F* ^2^ > 2σ(*F* ^2^)] = 0.029
*wR*(*F* ^2^) = 0.076
*S* = 1.11
3096 reflections
171 parameters
H-atom parameters constrained
Δρ_max_ = 0.25 e Å^−3^
Δρ_min_ = −0.26 e Å^−3^

### 

Data collection: *CrystalClear* (Rigaku, 2003 ▶); cell refinement: *CrystalClear*; data reduction: *CrystalClear*; program(s) used to solve structure: *SIR2004* (Burla *et al.*, 2005 ▶); program(s) used to refine structure: *SHELXL97* (Sheldrick, 2008 ▶); molecular graphics: *ORTEP-3 for Windows* (Farrugia, 2012 ▶); software used to prepare material for publication: *SHELXL97* and *Yadokari-XG 2009* (Kabuto *et al.*, 2009 ▶).

## Supplementary Material

Crystal structure: contains datablock(s) global, I. DOI: 10.1107/S1600536813002584/ds2224sup1.cif

Structure factors: contains datablock(s) I. DOI: 10.1107/S1600536813002584/ds2224Isup2.hkl

Supplementary material file. DOI: 10.1107/S1600536813002584/ds2224Isup3.cml

Additional supplementary materials:  crystallographic information; 3D view; checkCIF report

## Figures and Tables

**Fig. 1.**
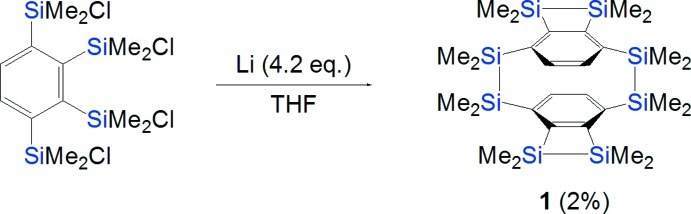
Synthesis of 1.

**Fig. 2.**
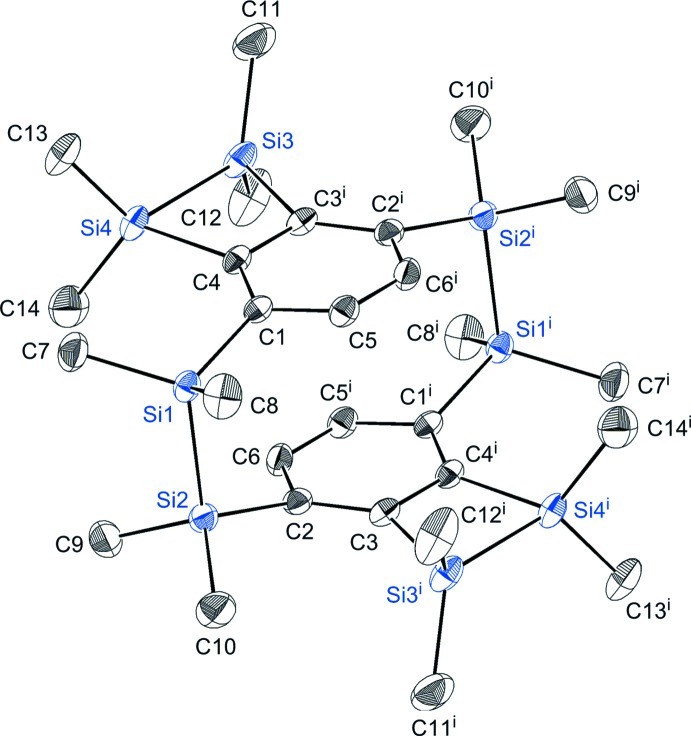
The molecular structure of 1, showing 50% probability displacement ellipsoids. [Symmetry code: (i) –x + 1, –y, –z + 1.]

**Fig. 3.**
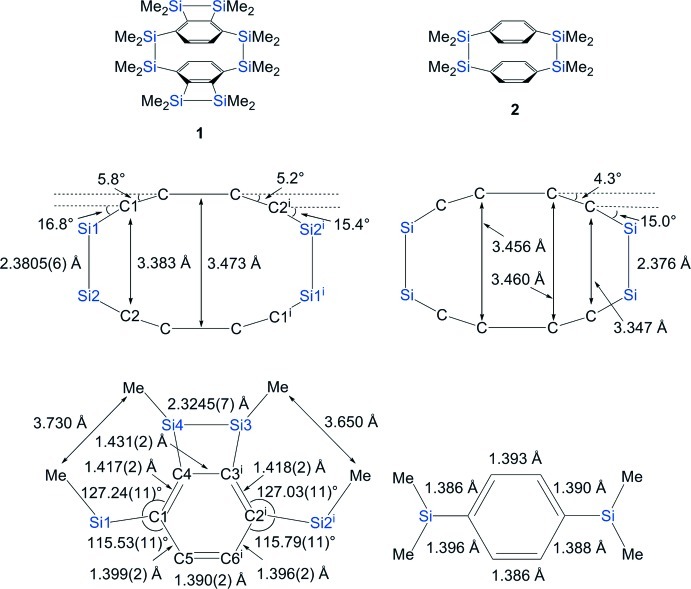
Comparison of the structures of 1 and 2.

**Fig. 4.**
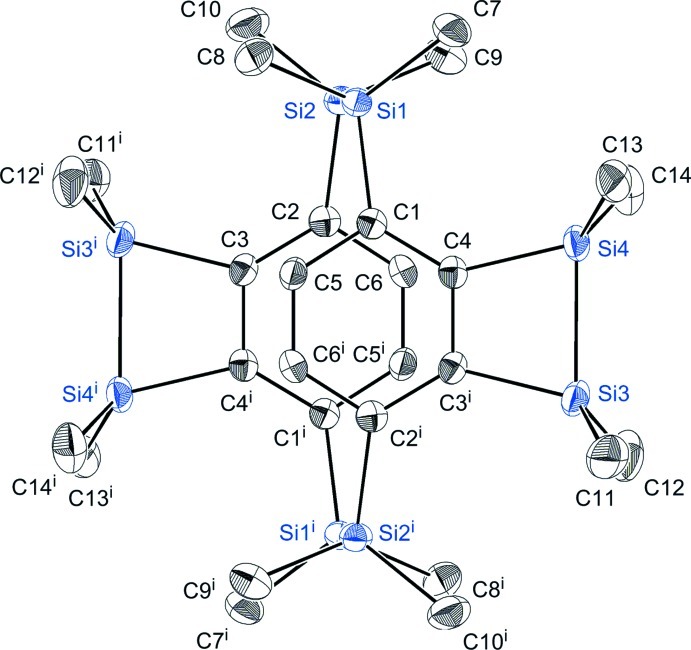
Top view of 1, showing 50% probability displacement ellipsoids. [Symmetry code: (i) –x + 1, –y, –z + 1.]

## supplementary crystallographic information

### Comment

Cyclophanes have been studied from the viewpoints of unique structures and interaction among aromatic rings. Some examples of cyclophanes bridged by silicon chains have so far been reported. [2.2]paracyclophane bridged by two 1,1,2,2-tetramethyldisilanylene chains has been synthesized, and its electronic properties have been reported (Sakurai *et al.*, 1986). Also, [2.2.2](1,3,5)cyclophane bridged by three 1,1,2,2-tetramethyldisilanylene chains has been synthesized (Sekiguchi *et al.*, 1989). Although cyclophanes bridged by silicon chains are attractive compounds, their studies have not further developed because of difficulty of synthesis. We report herein synthesis of a silicon-bridged [2.2]paracyclophane, in which two benzene rings are fused with 3,4-disilacyclobutene rings, and discuss the structural features of this compound.

The condensation of two molecules of 1,2,3,4-tetrakis(chlorodimethylsilyl)benzene with lithium in THF gave 1 in 2% yield (Fig. 1). The structure of 1 was determined by X-ray crystallography (Fig. 2). The molecule lies on an inversion center, and one half of the molecule corresponds to the asymmetric unit. Two 3,4-disila-1,2-benzocyclobutene rings are bridged by 1,1,2,2-tetramethyldisilanylene chains with an anti structure. The anti structure is favorable to avoid the steric hindrance among methyl groups on the 3,4-disilacyclobutene rings.

The benzene rings have a deformed structure due to fusion with a 3,4-disilacyclobutene ring (Fig. 3). The C—C bond is elongated in the order of C5—C6^i^ (1.390 (2) Å), C1—C5 (1.399 (2) Å) (or C2^i^—C6^i^ (1.396 (2) Å)), C1—C4 (1.417 (2) Å) (or C2^i^—C3^i^ (1.418 (2) Å)) and C4—C3^i^ (1.431 (2) Å). The Si1—C1—C4 and Si2^i^—C2^i^—C3^i^ bond angles are large (127.24 (11) and 127.03 (11)°, respectively), and the Si1—C1—C5 and Si2^i^—C2^i^—C6^i^ bond angles are small (115.53 (11) and 115.79 (11)°, respectively). As a result, two benzene rings are partially overlapped as shown in Fig. 4. This deformation of the bond angles is caused by the steric repulsion between the methyl groups on the Si1 and Si2^i^ atoms and the methyl groups on the Si3 and Si4 atoms. This steric repulsion also makes the Si3—Si4 bond short (2.3245 (7) Å) compared with the standard Si—Si bond (2.34 Å).

The benzene rings are also deformed by the cyclophane structure (Fig. 3). The benzene rings are not planar but have a slight boat conformation. The dihedral angle between the C4—C5—C6^i^—C3^i^ plane and the C1—C4—C5 (or C2^i^—C3^i^—C6^i^) plane is 5.8° (or 5.2°). The Si1—C1 and Si2^i^—C2^i^ bonds are further tilted from the C4—C5—C6^i^—C3^i^ plane with the angles of 16.8 and 15.4°. The distance between the C4—C5—C6^i^—C3^i^ and C3—C6—C5^i^—C4^i^ planes is 3.473 Å, and the distance between the C1 and C2 atoms is 3.383 Å. These structural features are similar to those of 1,1,2,2,9,9,10,10-octamethyl-1,2,9,10-tetrasila[2.2]paracyclophane (2) (Sakurai *et al.*, 1986) as shown in Fig. 3.

### Experimental

All operations except for Kugelrohr distillation were carried out in a glovebox. A mixture of 1,2,3,4-tetrakis(chlorodimethylsilyl)benzene (0.200 g, 0.446 mmol) and lithium (13.0 mg, 1.87 mmol) in THF (25 ml) was stirred at room temperature for 14 h. After removal of the solvent, the residue was dissolved in toluene, and insoluble materials were filtered off. The solvent was removed under reduced pressure. Kugelrohr distillation (300 °C/0.9 mm H g) of the residue gave a colorless solid. The solid was recrystallized from hexane to give 1 (3 mg, 2%) as colorless crystals. Single crystals were obtained from hexane by slow evaporation.

M.p.: 328–330 °C. ^1^H NMR (600 MHz, C_6_D_6_): *δ* 0.42 (s, 12H), 0.51 (s, 12H), 0.60 (s, 12H), 0.67 (s, 12H), 6.66 (s, 4H). ^13^C NMR (151 MHz, C_6_D_6_): *δ* -2.6, -1.3, -0.9, 136.0, 143.2, 160.2. ^29^Si NMR (119 MHz, C_6_D_6_): *δ* -18.1, -3.7. IR (KBr): 2960, 2930, 2900, 2850, 1260, 1250, 1100, 1080, 1020, 800, 750 cm^-1^. MS (EI, 70 eV): *m*/*z* 612 (*M*^+^, 29), 539 (23), 465 (18), 291 (15), 73 (100).

### Refinement

All hydrogen atoms were generated at calculated positions and refined as riding atoms with C—H = 0.95 (phenyl) or 0.98 (methyl) Å and *U*_iso_(H) = 1.2*U*_eq_(phenyl C) or 1.5*U*_eq_(methyl C).

### Crystal data

**Table:**

C_28_H_52_Si_8_	*F*(000) = 664
*M**_r_* = 613.42	*D*_x_ = 1.104 Mg m^−^^3^
Monoclinic, *P*2_1_/*n*	Melting point = 328–330 K
Hall symbol: -P 2yn	Mo *K*α radiation, λ = 0.71073 Å
*a* = 7.9801 (8) Å	Cell parameters from 11187 reflections
*b* = 18.9087 (14) Å	θ = 1.7–28.3°
*c* = 12.6222 (10) Å	µ = 0.31 mm^−^^1^
β = 104.2788 (9)°	*T* = 203 K
*V* = 1845.8 (3) Å^3^	Prism, colourless
*Z* = 2	0.25 × 0.25 × 0.25 mm

### Data collection

**Table:**

Rigaku R-AXIS IV Imaging Plate diffractometer	3096 independent reflections
Radiation source: rotating anode	3076 reflections with *I* > 2σ(*I*)
Graphite monochromator	*R*_int_ = 0.016
Detector resolution: 10.00 pixels mm^-1^	θ_max_ = 25.0°, θ_min_ = 2.0°
ω scans	*h* = −9→9
Absorption correction: multi-scan (*REQAB*; Jacobson, 1998)	*k* = −22→19
*T*_min_ = 0.927, *T*_max_ = 0.927	*l* = −15→15
9672 measured reflections	

### Refinement

**Table:**

Refinement on *F*^2^	Primary atom site location: structure-invariant direct methods
Least-squares matrix: full	Secondary atom site location: difference Fourier map
*R*[*F*^2^ > 2σ(*F*^2^)] = 0.029	Hydrogen site location: inferred from neighbouring sites
*wR*(*F*^2^) = 0.076	H-atom parameters constrained
*S* = 1.11	*w* = 1/[σ^2^(*F*_o_^2^) + (0.0349*P*)^2^ + 0.7873*P*] where *P* = (*F*_o_^2^ + 2*F*_c_^2^)/3
3096 reflections	(Δ/σ)_max_ = 0.021
171 parameters	Δρ_max_ = 0.25 e Å^−^^3^
0 restraints	Δρ_min_ = −0.26 e Å^−^^3^

### Special details

**Table:**

Geometry. All e.s.d.'s (except the e.s.d. in the dihedral angle between two l.s. planes) are estimated using the full covariance matrix. The cell e.s.d.'s are taken into account individually in the estimation of e.s.d.'s in distances, angles and torsion angles; correlations between e.s.d.'s in cell parameters are only used when they are defined by crystal symmetry. An approximate (isotropic) treatment of cell e.s.d.'s is used for estimating e.s.d.'s involving l.s. planes.
Refinement. Refinement of *F*^2^ against ALL reflections. The weighted *R*-factor *wR* and goodness of fit *S* are based on *F*^2^, conventional *R*-factors *R* are based on *F*, with *F* set to zero for negative *F*^2^. The threshold expression of *F*^2^ > 2σ(*F*^2^) is used only for calculating *R*-factors(gt) *etc.* and is not relevant to the choice of reflections for refinement. *R*-factors based on *F*^2^ are statistically about twice as large as those based on *F*, and *R*-factors based on ALL data will be even larger.

### Fractional atomic coordinates and isotropic or equivalent isotropic displacement parameters (Å^2^)

**Table:**

	*x*	*y*	*z*	*U*_iso_*/*U*_eq_	
Si1	0.18531 (5)	0.13135 (2)	0.45683 (3)	0.02369 (12)	
Si2	0.43507 (5)	0.17209 (2)	0.40399 (3)	0.02186 (12)	
Si3	0.50467 (6)	−0.03139 (2)	0.81537 (3)	0.02614 (12)	
Si4	0.36313 (6)	0.07548 (2)	0.76574 (3)	0.02856 (13)	
C1	0.24730 (19)	0.04152 (8)	0.52049 (12)	0.0218 (3)	
C2	0.58255 (18)	0.09258 (7)	0.41846 (11)	0.0202 (3)	
C3	0.58234 (19)	0.03950 (7)	0.33902 (11)	0.0209 (3)	
C4	0.33293 (19)	0.02681 (7)	0.63061 (12)	0.0212 (3)	
C5	0.2347 (2)	−0.01465 (8)	0.44684 (12)	0.0245 (3)	
C6	0.6830 (2)	0.07907 (8)	0.52380 (12)	0.0238 (3)	
C7	0.1117 (3)	0.19586 (9)	0.54917 (15)	0.0412 (4)	
C8	0.0026 (2)	0.12151 (10)	0.33167 (14)	0.0354 (4)	
C9	0.5452 (2)	0.24491 (9)	0.49722 (15)	0.0384 (4)	
C10	0.3789 (2)	0.21016 (9)	0.26238 (13)	0.0361 (4)	
C11	0.4144 (3)	−0.09537 (10)	0.90036 (13)	0.0402 (4)	
C12	0.7449 (2)	−0.02692 (11)	0.86886 (15)	0.0452 (5)	
C13	0.1573 (3)	0.09331 (11)	0.80715 (16)	0.0498 (5)	
C14	0.5025 (3)	0.15680 (10)	0.78452 (15)	0.0530 (6)	
H1	0.1681	−0.0086	0.3748	0.029*	
H2	0.6955	0.1149	0.5768	0.029*	
H3	0.0801	0.2402	0.5107	0.062*	
H4	0.2048	0.2041	0.6137	0.062*	
H5	0.0124	0.1767	0.5707	0.062*	
H6	−0.0946	0.0988	0.3512	0.053*	
H7	0.0401	0.0928	0.2781	0.053*	
H8	−0.0322	0.1678	0.3010	0.053*	
H9	0.6534	0.2570	0.4796	0.058*	
H10	0.5682	0.2291	0.5725	0.058*	
H11	0.4709	0.2862	0.4876	0.058*	
H12	0.3141	0.2536	0.2619	0.054*	
H13	0.3093	0.1765	0.2123	0.054*	
H14	0.4842	0.2201	0.2398	0.054*	
H15	0.4727	−0.1406	0.9023	0.060*	
H16	0.2916	−0.1016	0.8686	0.060*	
H17	0.4323	−0.0771	0.9741	0.060*	
H18	0.7727	−0.0130	0.9452	0.068*	
H19	0.7919	0.0076	0.8271	0.068*	
H20	0.7947	−0.0730	0.8620	0.068*	
H21	0.1841	0.1104	0.8819	0.075*	
H22	0.0906	0.0500	0.8019	0.075*	
H23	0.0906	0.1287	0.7591	0.075*	
H24	0.4378	0.1958	0.7440	0.079*	
H25	0.6045	0.1478	0.7578	0.079*	
H26	0.5373	0.1689	0.8615	0.079*	

### Atomic displacement parameters (Å^2^)

**Table:**

	*U*^11^	*U*^22^	*U*^33^	*U*^12^	*U*^13^	*U*^23^
Si1	0.0281 (2)	0.0210 (2)	0.0237 (2)	0.00390 (15)	0.00974 (17)	0.00293 (15)
Si2	0.0282 (2)	0.0175 (2)	0.0200 (2)	−0.00024 (15)	0.00618 (17)	0.00197 (14)
Si3	0.0354 (3)	0.0275 (2)	0.0153 (2)	−0.00299 (17)	0.00606 (18)	−0.00042 (15)
Si4	0.0461 (3)	0.0221 (2)	0.0199 (2)	−0.00391 (18)	0.01276 (19)	−0.00352 (16)
C1	0.0220 (8)	0.0228 (7)	0.0226 (7)	−0.0009 (5)	0.0093 (6)	0.0020 (6)
C2	0.0225 (8)	0.0200 (7)	0.0201 (7)	−0.0035 (5)	0.0088 (6)	0.0019 (5)
C3	0.0235 (8)	0.0218 (7)	0.0186 (7)	−0.0048 (5)	0.0075 (6)	0.0015 (5)
C4	0.0262 (8)	0.0200 (7)	0.0197 (7)	−0.0041 (5)	0.0100 (6)	−0.0012 (5)
C5	0.0278 (8)	0.0277 (8)	0.0170 (7)	0.0010 (6)	0.0036 (6)	0.0019 (6)
C6	0.0298 (9)	0.0226 (8)	0.0193 (7)	−0.0012 (6)	0.0067 (6)	−0.0031 (5)
C7	0.0567 (12)	0.0318 (9)	0.0415 (10)	0.0143 (8)	0.0239 (9)	0.0031 (8)
C8	0.0297 (9)	0.0378 (10)	0.0374 (9)	0.0032 (7)	0.0056 (7)	0.0081 (7)
C9	0.0510 (11)	0.0228 (8)	0.0390 (9)	−0.0052 (7)	0.0062 (8)	−0.0034 (7)
C10	0.0478 (11)	0.0330 (9)	0.0282 (9)	0.0084 (7)	0.0108 (8)	0.0100 (7)
C11	0.0630 (13)	0.0371 (10)	0.0242 (8)	−0.0023 (8)	0.0181 (8)	0.0047 (7)
C12	0.0414 (11)	0.0606 (13)	0.0306 (9)	−0.0043 (9)	0.0030 (8)	−0.0091 (8)
C13	0.0696 (14)	0.0514 (12)	0.0374 (10)	0.0154 (10)	0.0303 (10)	0.0010 (9)
C14	0.0894 (17)	0.0341 (10)	0.0328 (10)	−0.0244 (10)	0.0104 (10)	−0.0051 (8)

### Geometric parameters (Å, º)

**Table:**

Si1—Si2	2.3805 (6)	C7—H4	0.9700
Si1—C1	1.8919 (15)	C7—H5	0.9700
Si1—C7	1.8785 (17)	C8—H6	0.9700
Si1—C8	1.8762 (18)	C8—H7	0.9700
Si2—C2	1.8902 (15)	C8—H8	0.9700
Si2—C9	1.8824 (17)	C9—H9	0.9700
Si2—C10	1.8758 (16)	C9—H10	0.9700
Si3—Si4	2.3245 (7)	C9—H11	0.9700
Si3—C11	1.8746 (17)	C10—H12	0.9700
Si3—C12	1.871 (2)	C10—H13	0.9700
Si3—C3^i^	1.9067 (15)	C10—H14	0.9700
Si4—C4	1.9004 (15)	C11—H15	0.9700
Si4—C13	1.873 (2)	C11—H16	0.9700
Si4—C14	1.8781 (19)	C11—H17	0.9700
C1—C4	1.417 (2)	C12—H18	0.9700
C1—C5	1.399 (2)	C12—H19	0.9700
C2—C3	1.418 (2)	C12—H20	0.9700
C2—C6	1.396 (2)	C13—H21	0.9700
C3—C4^i^	1.431 (2)	C13—H22	0.9700
C5—C6^i^	1.390 (2)	C13—H23	0.9700
C5—H1	0.9400	C14—H24	0.9700
C6—H2	0.9400	C14—H25	0.9700
C7—H3	0.9700	C14—H26	0.9700
			
Si2—Si1—C1	105.08 (5)	H3—C7—H4	109.5
Si2—Si1—C7	112.03 (7)	H3—C7—H5	109.5
Si2—Si1—C8	109.07 (6)	H4—C7—H5	109.5
C1—Si1—C7	114.11 (7)	Si1—C8—H6	109.5
C1—Si1—C8	109.64 (7)	Si1—C8—H7	109.5
C7—Si1—C8	106.85 (9)	Si1—C8—H8	109.5
Si1—Si2—C2	105.07 (5)	H6—C8—H7	109.5
Si1—Si2—C9	110.84 (6)	H6—C8—H8	109.5
Si1—Si2—C10	111.80 (6)	H7—C8—H8	109.5
C2—Si2—C9	109.74 (8)	Si2—C9—H9	109.5
C2—Si2—C10	113.21 (7)	Si2—C9—H10	109.5
C9—Si2—C10	106.26 (8)	Si2—C9—H11	109.5
Si4—Si3—C11	119.05 (7)	H9—C9—H10	109.5
Si4—Si3—C12	116.39 (7)	H9—C9—H11	109.5
Si4—Si3—C3^i^	76.36 (5)	H10—C9—H11	109.5
C11—Si3—C12	109.08 (9)	Si2—C10—H12	109.5
C11—Si3—C3^i^	116.01 (7)	Si2—C10—H13	109.5
C12—Si3—C3^i^	117.06 (7)	Si2—C10—H14	109.5
Si3—Si4—C4	76.50 (5)	H12—C10—H13	109.5
Si3—Si4—C13	118.85 (7)	H12—C10—H14	109.5
Si3—Si4—C14	116.39 (8)	H13—C10—H14	109.5
C4—Si4—C13	114.32 (8)	Si3—C11—H15	109.5
C4—Si4—C14	116.73 (8)	Si3—C11—H16	109.5
C13—Si4—C14	110.50 (11)	Si3—C11—H17	109.5
Si1—C1—C4	127.24 (11)	H15—C11—H16	109.5
Si1—C1—C5	115.53 (11)	H15—C11—H17	109.5
C4—C1—C5	116.18 (13)	H16—C11—H17	109.5
Si2—C2—C3	127.03 (11)	Si3—C12—H18	109.5
Si2—C2—C6	115.79 (11)	Si3—C12—H19	109.5
C3—C2—C6	116.36 (13)	Si3—C12—H20	109.5
Si3^i^—C3—C2	135.73 (11)	H18—C12—H19	109.5
Si3^i^—C3—C4^i^	103.43 (10)	H18—C12—H20	109.5
C2—C3—C4^i^	120.82 (13)	H19—C12—H20	109.5
Si4—C4—C1	135.16 (11)	Si4—C13—H21	109.5
Si4—C4—C3^i^	103.70 (10)	Si4—C13—H22	109.5
C1—C4—C3^i^	121.09 (13)	Si4—C13—H23	109.5
C1—C5—C6^i^	122.44 (14)	H21—C13—H22	109.5
C1—C5—H1	118.8	H21—C13—H23	109.5
C6^i^—C5—H1	118.8	H22—C13—H23	109.5
C2—C6—C5^i^	122.46 (13)	Si4—C14—H24	109.5
C2—C6—H2	118.8	Si4—C14—H25	109.5
C5^i^—C6—H2	118.8	Si4—C14—H26	109.5
Si1—C7—H3	109.5	H24—C14—H25	109.5
Si1—C7—H4	109.5	H24—C14—H26	109.5
Si1—C7—H5	109.5	H25—C14—H26	109.5
			
C1—Si1—Si2—C2	11.34 (7)	C12—Si3—Si4—C4	113.08 (8)
C1—Si1—Si2—C9	−107.13 (8)	C12—Si3—Si4—C13	−136.36 (10)
C1—Si1—Si2—C10	134.52 (8)	C12—Si3—Si4—C14	−0.33 (10)
C7—Si1—Si2—C2	135.78 (8)	C3^i^—Si3—Si4—C4	−0.64 (6)
C7—Si1—Si2—C9	17.31 (9)	C3^i^—Si3—Si4—C13	109.93 (9)
C7—Si1—Si2—C10	−101.04 (9)	C3^i^—Si3—Si4—C14	−114.04 (8)
C8—Si1—Si2—C2	−106.14 (8)	Si3—Si4—C4—C1	178.26 (16)
C8—Si1—Si2—C9	135.39 (9)	Si3—Si4—C4—C3^i^	0.85 (8)
C8—Si1—Si2—C10	17.04 (9)	C13—Si4—C4—C1	62.41 (17)
Si2—Si1—C1—C4	85.99 (13)	C13—Si4—C4—C3^i^	−115.00 (11)
Si2—Si1—C1—C5	−81.76 (11)	C14—Si4—C4—C1	−68.73 (18)
C7—Si1—C1—C4	−37.12 (16)	C14—Si4—C4—C3^i^	113.86 (12)
C7—Si1—C1—C5	155.13 (12)	Si1—C1—C4—Si4	21.9 (2)
C8—Si1—C1—C4	−156.92 (13)	Si1—C1—C4—C3^i^	−160.99 (11)
C8—Si1—C1—C5	35.33 (13)	C5—C1—C4—Si4	−170.37 (12)
Si1—Si2—C2—C3	86.84 (12)	C5—C1—C4—C3^i^	6.7 (2)
Si1—Si2—C2—C6	−82.38 (11)	Si1—C1—C5—C6^i^	162.33 (12)
C9—Si2—C2—C3	−153.95 (13)	C4—C1—C5—C6^i^	−6.8 (2)
C9—Si2—C2—C6	36.83 (13)	Si2—C2—C3—Si3^i^	18.9 (2)
C10—Si2—C2—C3	−35.43 (15)	Si2—C2—C3—C4^i^	−163.08 (11)
C10—Si2—C2—C6	155.35 (11)	C6—C2—C3—Si3^i^	−171.94 (12)
C11—Si3—Si4—C4	−113.06 (8)	C6—C2—C3—C4^i^	6.1 (2)
C11—Si3—Si4—C13	−2.49 (11)	Si2—C2—C6—C5^i^	164.24 (12)
C11—Si3—Si4—C14	133.53 (10)	C3—C2—C6—C5^i^	−6.2 (2)

Symmetry code: (i) −*x*+1, −*y*, −*z*+1.
